# Advanced Image‐Guidance and Surgical‐Navigation Techniques for Real‐Time Visualized Surgery

**DOI:** 10.1002/advs.202509294

**Published:** 2025-09-23

**Authors:** Xiaoxiao Fan, Xiaolong Liu, Qiming Xia, Guoqiao Chen, Jiaxi Cheng, Zhaoqi Shi, Yifeng Fang, Parikshit Asutosh Khadaroo, Jun Qian, Hui Lin

**Affiliations:** ^1^ Department of General Surgery Sir Run Run Shaw Hospital School of Medicine Zhejiang University Hangzhou 310016 China; ^2^ Neuropsychiatry and ECT Department The Royal Melbourne Hospital 300 Grattan Street Parkville Victoria Australia; ^3^ State Key Laboratory of Extreme Photonics and Instrumentation International Research Center for Advanced Photonics Centre for Optical and Electromagnetic Research College of Optical Science and Engineering Zhejiang University Hangzhou China; ^4^ College of Biomedical Engineering and Instrument Science Zhejiang University Hangzhou China; ^5^ Zhejiang Engineering Research Center of Cognitive Healthcare Sir Run Run Shaw Hospital School of Medicine Zhejiang University Hangzhou 310016 China

**Keywords:** biomedical engineering, precision surgery, surgical navigation, visualized surgery

## Abstract

Surgical navigation is a rapidly evolving multidisciplinary system that plays a crucial role in precision medicine. Surgical‐navigation systems have substantially enhanced modern surgery by improving the precision of resection, reducing invasiveness, and enhancing patient outcomes. However, clinicians, engineers, and professionals in other fields often view this field from their own perspectives, which usually results in a one‐sided viewpoint. This article aims to provide a thorough overview of the recent advancements in surgical‐navigation systems and categorizes them on the basis of their unique characteristics and applications. Established techniques (e.g., radiography, intraoperative computed tomography [CT], magnetic resonance imaging [MRI], and ultrasound) and emerging technologies (e.g., photoacoustic imaging and near‐infrared [NIR]‐II imaging) are systematically analyzed, highlighting their underlying mechanisms, methods of use, and respective advantages and disadvantages. Despite substantial progress, the existing navigation systems face challenges, including limited accuracy, high costs, and extensive training requirements for surgeons. Addressing these limitations is crucial for widespread adoption of these technologies. The review emphasizes the need for developing more intelligent, minimally invasive, precise, personalized, and radiation‐free navigation solutions. By integrating advanced imaging modalities, machine learning algorithms, and real‐time feedback mechanisms, next‐generation surgical‐navigation systems can further enhance surgical precision and patient safety. By bridging the knowledge gap between clinical practice and engineering innovation, this review not only provides valuable insights for surgeons seeking optimal navigation strategies, but also offers engineers a deeper understanding of clinical application scenarios.

## Graphical abstract

This review comprehensively summarizes advances in surgical navigation, covering both established and emerging intraoperative imaging technologies. It highlights mechanisms, advantages, limitations, and clinical applications, while emphasizing interdisciplinary collaboration. By integrating imaging modalities with intelligent algorithms, next‐generation systems aim to enhance surgical precision, minimize invasiveness, and improve patient outcomes.

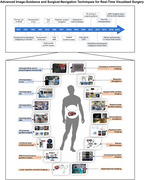

## 1 Introduction

Surgery is an important therapeutic strategy for combating human diseases, including cancer, trauma, and deformities. In traditional surgery, surgeons primarily rely on visual and tactile modalities to sense different tissues. However, visual modalities are primarily affected by the minimal chromatic aberration and substantial scattering in human tissues. During surgical procedures, surgeons primarily aim to identify diseased lesions and avoid injury to important normal organs.^[^ ^1^ ^]^ However, the chromatic differences between the diseased and normal tissues may be minimal. Thus, surgeons cannot easily distinguish between these two types of tissues with the naked eye. Diseased lesions may also be embedded in normal tissues and are difficult to observe directly.^[^ ^2^ ^]^ In addition, the localization of implants to ensure the presence of implants or foreign bodies is another imperative demand in clinical practice. Meanwhile, the tactile sense is gradually being lost with the development of laparoscopic and robotic surgery.^[^ ^3^ ^]^ Surgical‐navigation systems have been established to fulfill this need. The requirements for navigation techniques in surgery can be roughly classified into three categories: tracing diseased tissues, distinguishing normal tissues, and locating implants.

With advancements in our understanding of anatomy and the development of novel surgical techniques, surgery has transitioned from reliance on empiricism to a more precise approach. The concept of precision surgery was proposed in 2013 by Jiahong Dong.^[^ ^4^ ^]^ This concept emphasizes three important issues for surgery: maximizing the resection of all diseased lesions, maximizing the preservation of normal tissues, and damage control.^[^ ^5^ ^]^ Various surgical‐navigation systems have been developed to address these issues. In this regard, scientists, engineers, and surgeons should work together to develop the field of surgical navigation and improve patient outcomes. Higher requirements must be met for more precise and complex navigation systems. The fundamental goals of surgical navigation are to solve the challenges posed by highly similar chromatic differences between diseased and normal tissues and spatial localization.^[^ ^6^ ^]^

Surgical‐navigation technology is a multidisciplinary system that integrates fields such as medicine, optical science, computer technology, robotics, spatial positioning technology, virtual reality (VR) interaction, and custom manufacturing.^[^ ^7^, ^8^ ^]^ On the basis of the detection methods, navigation technologies can be roughly classified into three types: optical navigation technologies, non‐optical electromagnetic technologies, and other technologies.^[^ ^9^ ^]^ Optical technologies include fluorescence imaging, photoacoustic imaging (PAI), and Raman spectroscopy (RS). These technologies can provide real‐time and high‐resolution images during surgery and represent the mainstream technology for operations. However, the main limitation of these technologies is their limited penetration depth. Non‐optical electromagnetic technologies, including radiography, computed tomography (CT), and radionuclide imaging, can complement optical technology. However, these methods usually yield static images on viewing screens away from the surgical field. The third type, other technologies, includes methods that are not based on electromagnetic waves, such as intraoperative ultrasound, magnetic resonance imaging (MRI), 3D (3D)‐printing technology, and VR‐based technologies.

The development of intraoperative navigation technology has been ongoing for more than 80 years. Commonly used intraoperative medical image navigation techniques include radiography, CT, MRI, and ultrasound, which have been safely used in clinical practice for decades. By displaying real‐time images, the anatomical tissues are highlighted, providing surgeons with a clearer view of the operation. More surgical‐navigation techniques are gradually being introduced to facilitate the operation process. Those techniques render the anatomical structure transparent and provide surgeons with a pair of “perspective glasses” to distinguish different tissues. In addition, real‐time guidance during surgery is an important aspect of surgical‐navigation systems. On the basis of this aspect, surgical‐navigation techniques can be divided into real‐time and non‐real‐time techniques. Typical real‐time techniques include fluorescence and radionuclide imaging, while typical non‐real‐time techniques include intraoperative CT or MRI and radiography. The pattern of surgery transition from “blind surgery” to “real‐time visualized surgery.”

The objective of this study was to comprehensively review the current and prospective intraoperative technologies used in clinical surgical navigation. Surgeons, who primarily focus on enhancing surgical techniques, often lack familiarity with engineering methodologies. Notably, no existing review offers surgeons a holistic view of the navigational landscape. Consequently, this study will aid surgeons in identifying and applying suitable navigation techniques in clinical practice. Conversely, engineers, who are engrossed in advancing their technological research, often lack comprehensive insights into clinical application scenarios. Therefore, this review can serve as a crucial reference for engineers by offering them valuable medical perspectives. By synthesizing various clinical scenarios and technology specifics, this review aims to foster interdisciplinary collaboration, ultimately propelling advancements in clinical surgical precision.

**Figure 1:**
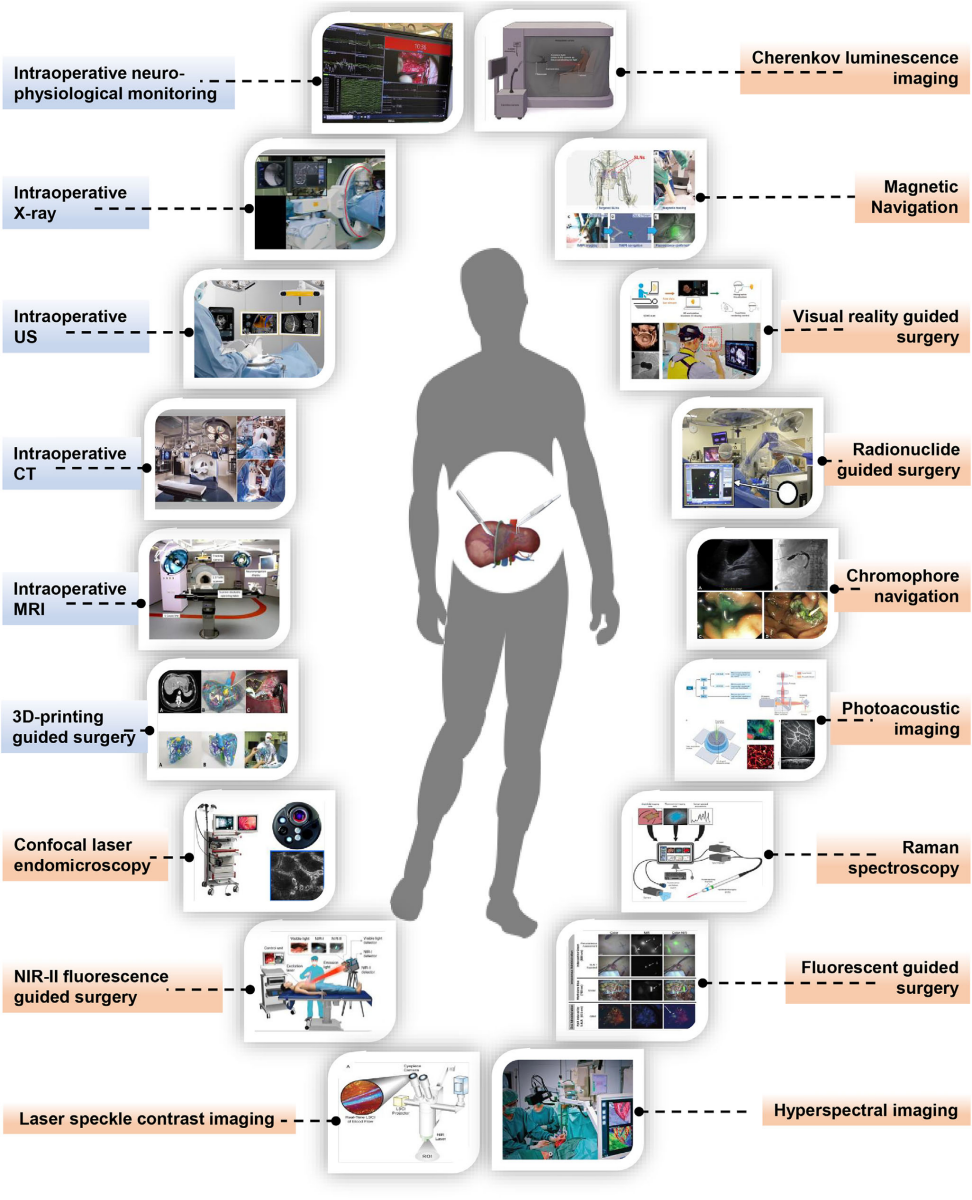
Overview of surgical‐navigation techniques used clinically. The technologies in the blue boxes represent non‐real‐time surgical‐navigation technologies, while those in the orange boxes represent real‐time surgical‐navigation technologies.

**Figure 2:**
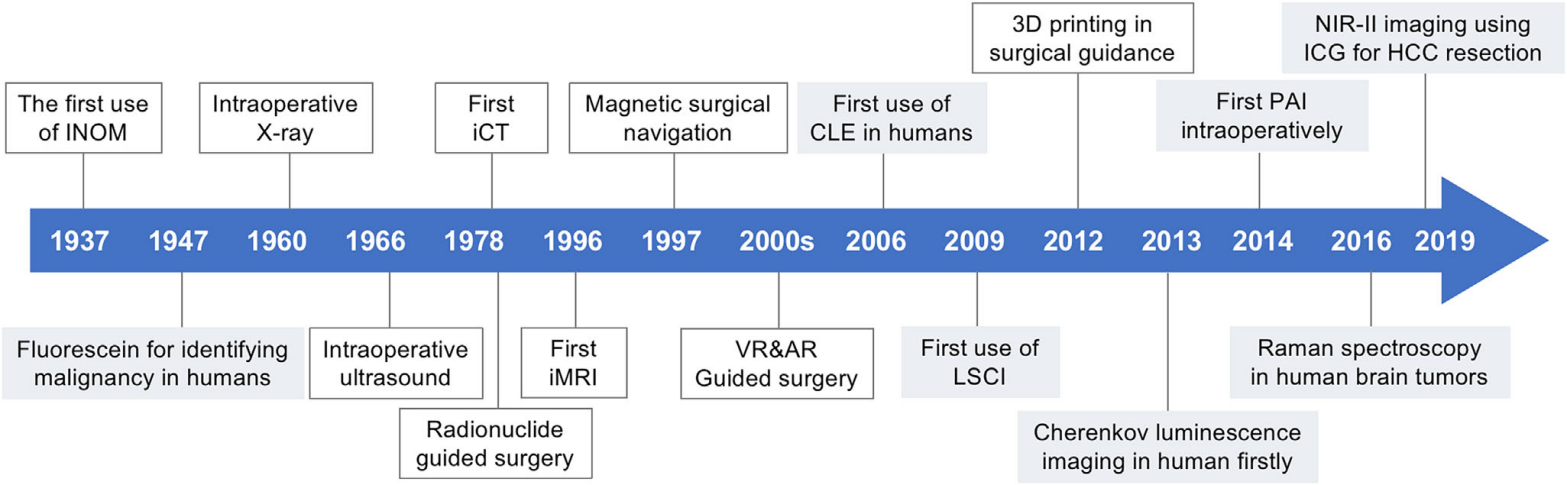
The timeline of the first clinical use of the surgical‐navigation techniques listed in Figure 1.

We conducted a comprehensive literature search using PubMed, IEEE Xplore, and Google Scholar. The search terms included “intraoperative navigation,” “surgical navigation,” and “clinician‐engineer collaboration.” Boolean operators (AND, OR, and NOT) were used to refine search results. We reviewed articles published between 1900 and 2024 and filtered the important and representative articles in each field. Data pertaining to the study design, sample size, methodologies, and outcomes were extracted, and related research was identified. This review encompasses a spectrum of surgical‐navigation technologies, including established methods and emerging innovations currently undergoing preliminary clinical trials (**Figure** **1**). The timeline of the initial clinical implementation of these techniques is shown in **Figure** **2**. By bridging the knowledge gap between clinical practice and engineering innovation, this study not only provides valuable insights for surgeons seeking optimal navigation strategies, but also offers engineers a deeper understanding of clinical application scenarios. This collaborative approach is pivotal for advancing the field of surgical‐navigation technology and, ultimately, driving improvements in surgical outcomes.

## 2 Non‐Real‐Time Surgical‐Navigation Techniques

### 2.1 Intraoperative Radiography

The invention of X‐rays was a milestone in medicine. Based on the different absorptions of X‐ray photons in tissues with different chemical compositions, such as calcium‐rich bone and carbon‐rich soft tissue, a 2D image of the human body can be created to reveal normal tissues and lesions.^[^ ^10^ ^]^ Intraoperative X‐ray technology has been long and widely used in orthopedic surgery due to its advantages such as convenient operation, repeated shooting, clear display of the position of bones and implants (pedicle screws, plates, etc.), and demonstration of anatomical stability.^[^ ^11^, ^12^ ^]^ For example, during resection of sacrococcygeal teratomas, intraoperative pelvic radiographs are used to locate boundaries and ensure total coccyx resection to reduce the risk of recurrence.^[^ ^13^ ^]^ This technology has also been used during acetabular surgery to achieve fracture reduction and accurate screw placement to reduce the risk of reoperation.^[^ ^14^ ^]^

However, the use of radiography during surgery is associated with some problems. First, the intraoperative use of X‐rays may pose a serious risk of occupational radiation exposure, especially in comparison with continuous fluoroscopy.^[^ ^15^, ^16^ ^]^ Second, the C‐arm, although normally worn with an aseptic protective sleeve, may be a potential source of contamination during repeated movements.^[^ ^17^ ^]^ In addition, X‐rays can only show 2D images of a single plane. Therefore, the bulky C‐arm has to be frequently repositioned to obtain photographs from different angles.^[^ ^18^ ^]^

### 2.2 Intraoperative Ultrasound

Intraoperative ultrasonography (IOUS) has gradually become an indispensable tool in various surgical fields since its introduction approximately 40 years ago.^[^ ^19^ ^]^ Ultrasonic probes have high frequency and small size, which allow them to be placed directly on the surface of organs for detection. This overcomes the limitations of in vitro ultrasound and increases the resolution and scanning range, thereby improving the detection rate for small lesions. The advantages of IOUS, including its real‐time, convenient, flexible, safe, non‐invasive, and accurate positioning capabilities along with its low cost and ability to be used for repeated examinations, have drawn the attention of an increasing number of surgeons.^[^ ^20^ ^]^ Owing to its widespread use, IOUS has played an essential role in many surgical procedures.

Studies have demonstrated that IOUS‐guided resection of hepatocellular carcinoma not only ensures a negative resection margin but also preserves crucial ducts (bile ducts and blood vessels) surrounding the tumor, thereby reducing the extent of hepatectomy.^[^ ^21^ ^]^ Additionally, IOUS has been found to increase the detection rate of lesions and improve the accuracy of diagnosis and treatment.^[^ ^22^ ^]^ In neurosurgery, IOUS technology has played a crucial role in accurately identifying and defining the boundaries of intracranial tumors. This approach enables immediate monitoring of the surgical process, guiding surgeons to remove lesions efficiently and precisely, leading to maximal tumor removal and minimal brain damage.^[^ ^23^ ^]^

Since ultrasound examination is contact‐based, tumor resection and ultrasound examination cannot be performed simultaneously. With advancements in technology, a clinical strategy has been developed to co‐register preoperative imaging data with intraoperative ultrasound.^[^ ^24^ ^]^ This enables precise surgical treatment of various conditions, including breast cancer, liver cancer, nervous system diseases, and orthopedic diseases, through 3D reconstruction and surgical path guidance.^[^ ^24^, ^25^, ^26^ ^]^ IOUS can be reasonably expected to achieve higher positioning accuracy with further improvements in ultrasonic elastic imaging, 3D visualization, contrast‐enhanced ultrasound, and other technologies, thus creating a wide range of application prospects.^[^ ^27^ ^]^

### 2.3 Intraoperative Computed Tomography

In comparison with radiography, intraoperative CT (iCT) offers the advantages of high tissue resolution and 3D imaging capability.^[^ ^18^ ^]^ Patients can complete CT imaging examinations during surgery in the operating room, avoiding the injury caused by multiple transports, greatly shortening the time window of treatment, and improving the accuracy of surgical treatment. Through compatible connections between the CT workstation and navigation systems based on the Digital Imaging and Communications in Medicine (DICOM) standard, CT image data obtained during surgery can be immediately sent to the navigation system to guide the surgery.^[^ ^28^ ^]^

iCT technology was introduced in modern neurosurgery and spinal surgery in the early 1980s.^[^ ^29^ ^]^ With the development of O‐arms and a variety of hardware and software, the image quality in iCT has improved dramatically, facilitating routine applications in a wide range of surgeries.^[^ ^30^ ^]^ For instance, iCT (O‐arm surgical imaging system) imaging guidance in conjunction with a navigation system has been shown to improve the accuracy of spinal instrumentation placement (pedicle screws, etc.) and effectively navigate surgery of complex bone anatomy altered by tumor growth and abnormal bone growth.^[^ ^31^, ^32^, ^33^ ^]^ In neurosurgery, iCT has been used for neuronavigation in surgical resection of complex anatomical brain tumors and for intraoperative angiography of cerebrovascular diseases.^[^ ^34^, ^35^ ^]^

### 2.4 Intraoperative Magnetic Resonance Imaging

Owing to the limitations of iCT imaging, such as the potential for radiation damage and poor contrast of soft tissues, intraoperative MRI (iMRI) technology, which has natural advantages to address these limitations, became a key development direction for intraoperative imaging in the 1990s. After the performance of the first iMRI‐guided surgery at the Department of Neurosurgery, Brigham Hospital, in June 1995, iMRI has been widely used in the resection of intracranial space‐occupying lesions, functional neurosurgery, directional puncture biopsy, and other fields owing to its advantages in intraoperative imaging, timely correction of intraoperative brain displacement errors, accurate guidance of surgery, and puncture operation.^[^ ^36^, ^37^, ^38^ ^]^

A cohort study was conducted on 116 cases involving glioma resection under the guidance of iMRI, visual examination, and 5‐amino‐acetylacetylic acid staining, and the results showed that the iMRI‐guided surgery could significantly improve the complete resection rate of tumors, thereby improving the prognosis of patients and extending the survival time.^[^ ^39^ ^]^ In addition, iMRI‐guided resection of invasive pituitary tumors can reduce the incidence of injuries to the optic nerve, internal carotid artery, and other important structures, greatly reducing the incidence of postoperative complications.^[^ ^40^ ^]^ iMRI‐based neural functional navigation technology has also been established to assist surgeons in maximizing the resection of tumors adjacent to important cerebral language areas or nerve conduction tracts, reducing neurological injury, and promoting postoperative neurological recovery.^[^ ^41^, ^42^ ^]^

However, this technology also has certain limitations. A better understanding of these limitations is important for the future clinical popularization of iMRI. First, all surgical tools, including respiratory anesthesia equipment and monitoring instruments, expected to be used in the MRI environment should be “MRI‐safe” medical instruments, which requires a special operating room.^[^ ^43^ ^]^ Both iMRI instruments and operating room‐compatible devices incur high costs.^[^ ^44^ ^]^ Additionally, the image quality of iMRI can be negatively affected by various factors, including surgical procedures and radiofrequency noise from undershielded electronics.^[^ ^45^ ^]^ Continued improvements in the imaging performance of iMRI and increasing the adaptability of iMRI to a normal operative environment are essential to address these limitations.

### 2.5 3D Printing‐Guided Surgery

3D printing technology is advancing at an exponential rate, enabling the data from medical imaging modalities, including CT, MRI, and 3D ultrasound, to be converted into handheld anatomical models of organs that can be rapidly and intuitively identified, analyzed, and manipulated in the surgical environment.^[^ ^46^ ^]^ This approach is effective for planning and simulating surgical procedures, identifying anatomical markers, and observing potential invasion of vascular and other vital organs in patients with cancer. 3D‐printed models have been widely used in many surgeries, including orthopedics, otorhinolaryngology, general surgery, and thoracic surgery.^[^ ^46^, ^47^, ^48^, ^49^ ^]^

In complex liver surgery, such as resection of liver cancer with portal vein invasion or multiple metastases, 3D printing facilitates intraoperative navigation to direct surgical pathways, detect metastases, and improve excision planning.^[^ ^50^ ^]^ The benefits of 3D‐printed models for intraoperative path planning and guidance in orthopedic surgery are clear, particularly in areas such as deformity correction and fracture fixation.^[^ ^51^ ^]^ In addition, 3D printing technology is frequently used in otolaryngology and head and neck surgery to transform the complex 3D anatomical structure of the sinuses and nasal skull base into a visual model, making the operation more intuitive and convenient and greatly shortening the operation time.^[^ ^52^, ^53^ ^]^

**Figure 3:**
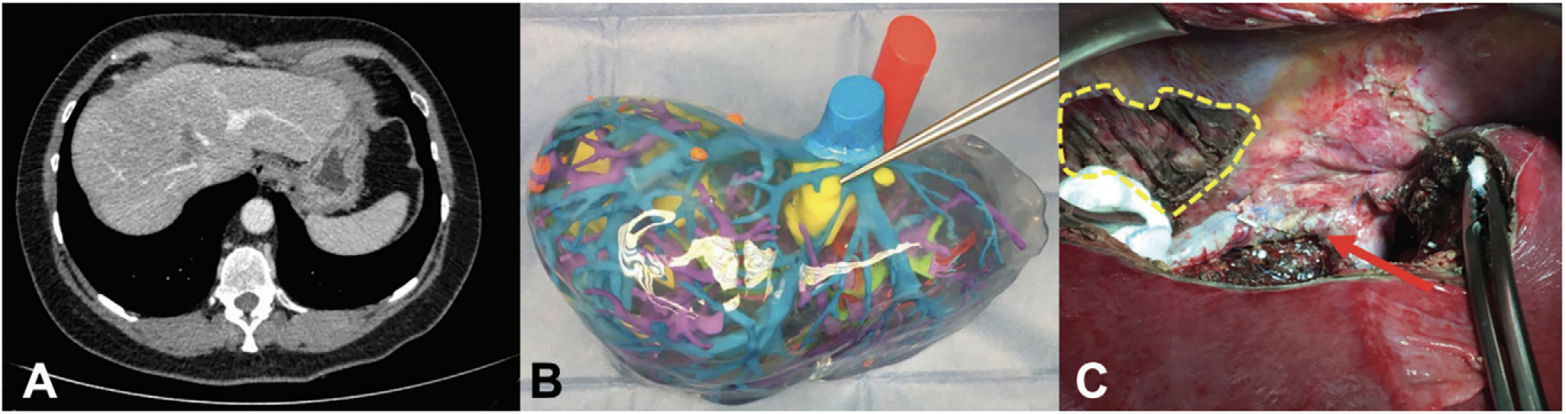
3D printing‐guided liver surgery. A) Preoperative CT scan of a patient with colorectal liver metastases. B) 3D printing of a liver model relative to the patient. C) Post‐resection reconstruction of the right hepatic vein with a peritoneal patch (indicated by the red arrow) taken from the diaphragm (highlighted by the yellow line) (Reproduced with permission). Copyright 2021, Lippincott Williams and Wilkins Ltd.^[^ ^50^ ^]^

The use of 3D printing technology for surgical navigation remains in its infancy.^[^ ^54^ ^]^ Although this technology can provide technical support for simulating surgery and designing intraoperative navigation, its clinical application has certain limitations, including advanced technical requirements, hardware and software requirements, and high costs, which limit its widespread use in hospitals (**Figure** **3**).^[^ ^50^ ^]^

### 2.6 Intraoperative Neurophysiological Monitoring

Intraoperative neurophysiological monitoring (IONM) utilizes a range of neurophysiological techniques to monitor the integrity of the nervous system during complex surgical procedures.^[^ ^55^ ^]^ Over time, IONM has evolved into an indispensable component of clinical operations that is aimed at safeguarding nerve function, reducing nerve injury, and enhancing surgical outcomes. While modern medical surgery benefits greatly from various imaging modalities that facilitate precise anatomical visualization, IONM offers a distinct perspective by assessing nervous system function. It provides an objective evaluation of nervous system integrity across diverse surgical specialties such as neurosurgery, orthopedics, and head and neck surgery.^[^ ^56^, ^57^, ^58^ ^]^ By providing surgeons with reliable real‐time information, IONM contributes to smoother and safer surgical interventions.

In 1937, Penfield and Boldrey first reported the use of IONM in the resection of epileptogenic foci in patients with epilepsy.^[^ ^59^ ^]^ Since then, IONM has been sporadically used in neurosurgery. Until the 1970s, IONM was routinely used to monitor cerebral ischemia and hypoxia during carotid endarterectomy (CEA).^[^ ^60^ ^]^ Concurrently, spinal cord monitoring techniques began to develop, with assessment of somatosensory‐evoked potentials (SEPs) emerging as a technique for intraoperative monitoring of sensory conduction pathway function during craniocerebral and spinal surgery.^[^ ^61^ ^]^ Recently, the use of motor‐evoked potentials (MEPs) has further advanced the intraoperative monitoring of motor function, contributing to the refinement of intraoperative electrophysiological monitoring of the nervous system.^[^ ^62^ ^]^

The integration of multiple neurophysiological monitoring techniques can synergistically enhance their efficacy, validate findings across methods, facilitate real‐time assessment of neurological function, and provide guidance during surgical procedures. This holistic approach can help mitigate potential intra‐ and postoperative neurological complications, optimizing the accuracy and thoroughness of lesion resection while prioritizing patient safety.

## 3 Real‐Time Surgical‐Navigation Techniques

### 3.1 Chromophore Navigation

Some organic compound molecules that contain unsaturated bond groups, known as chromophores, can selectively absorb a certain wavelength of light in the ultraviolet and visible regions (200–800 nm) to make the material appear in unique colors. These compounds, which include methylene blue (MB), isosulfan blue, indigo carmine, and gentian violet, have specific colors and can be useful tools during surgery, helping surgeons identify the locations of lesions with the naked eye and perform precise excisions.^[^ ^63^ ^]^

MB is widely used as a chromophore dye in surgical operations because of its low price, rapid dispersion, small molecular weight, high biological safety, simple operation during the surgery process, and lack of the need for special equipment. In the surgical treatment of primary hyperparathyroidism, intraoperative MB perfusion (IMBI) helped distinguish normal and proliferative glands and locate ectopic glands, which greatly reduces the time required for bilateral neck exploration and improves surgical accuracy.^[^ ^64^ ^]^ In one case report, a patient with unexplained gastrointestinal bleeding was successfully diagnosed and underwent segmental enterectomy under intraoperative MB navigation.^[^ ^65^ ^]^ In addition, MB is most frequently used for intraoperative lymph node tracing, such as sentinel lymph node biopsy (SLNB) and mapping in breast cancer, gastric cancer, colorectal cancer, and endometrial cancer.^[^ ^66^, ^67^, ^68^, ^69^ ^]^

However, chromophore dye‐based navigation has certain limitations. Isosulfan blue can induce human allergic reactions, which generally manifest as dizziness and nausea and can lead to anaphylactic shock;^[^ ^70^ ^]^ in contrast, MB is relatively safer.^[^ ^71^ ^]^ In addition, these dyes are easily dispersed in tissues, which is not conducive to the accurate identification of lesions and tends to cause false‐positive results. The detection rate of sentinel lymph nodes (SLNs) is only 60%–70% when MB is used as the tracer,^[^ ^72^ ^]^ implying the need for further improvement to improve the accuracy of this approach.

### 3.2 Magnetic Navigation

The magnetic navigation system (MNS) is mostly commonly used in neurosurgery and functions like a “GPS for the brain,” providing surgeons with a “shortcut” to treating diseases. The basic workflow and principle of the MNS are as follows. First, the image information is collected before surgery, and the navigation system is fitted through 3D reconstruction. The magnetic navigation probe can then be used to point at the surgical site during the operation, and the 3D mapping between the probe and body structure can be virtually displayed on the navigation screen.^[^ ^73^ ^]^ This function allows the surgeon to clearly identify the proximity of the lesion to surrounding important structures and plan the surgical path. By establishing local magnetic fields and converting them into 3D spatial coordinates, MNS significantly improves surgical accuracy and safety.^[^ ^74^ ^]^

With recent advancements in magnetic navigation technology, its clinical application has gradually expanded to bronchoscopy, spinal orthopedics, neurosurgery, and other fields.^[^ ^75^, ^76^ ^]^ Many clinical trials have proven that MNS is of high value in surgical operations such as resection of intracranial tumors in neurosurgery, especially large gliomas with unclear intracranial boundaries; radiofrequency ablation for arrhythmias; vertebral nail implantation in spinal surgery, joint replacement and other complex orthopedic operations; and radical resection of lung tumors assisted by electromagnetic bronchoscopy.^[^ ^75^, ^77^, ^78^, ^79^, ^80^ ^]^

Electromagnetic navigation systems have the following advantages: no optical obstruction, convenient use during operations, small size, and portability, which greatly alleviate the pressure of insufficient operating room space.^[^ ^81^ ^]^ Additionally, the MNS yields real‐time images that can be tracked continuously without repositioning.^[^ ^82^ ^]^ With the miniaturization and improved precision of magnetic field transmitters, MNS is expected to become a part of a new generation of navigation technologies.

### 3.3 Visual Reality‐Guided Surgery

**Figure 4:**
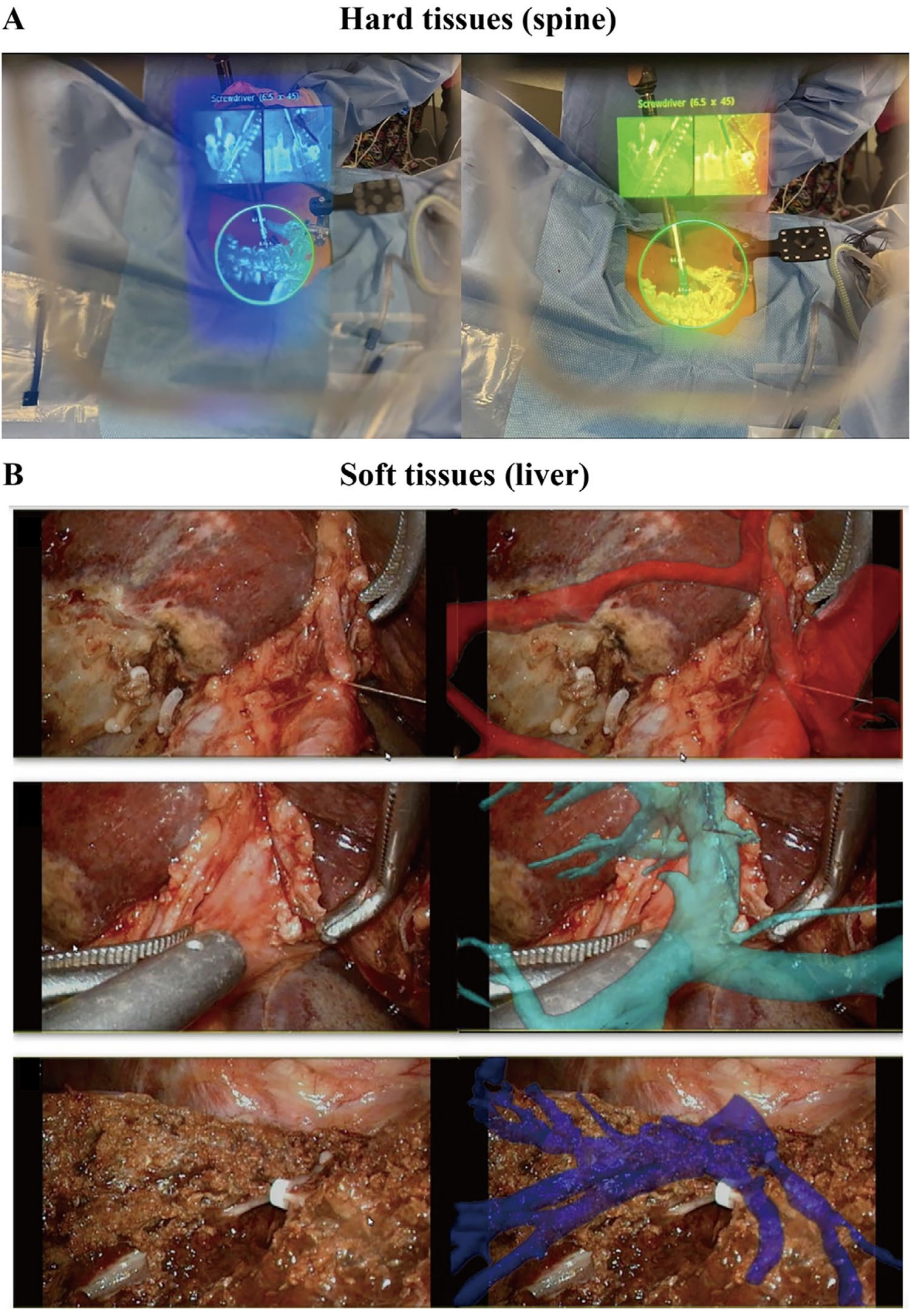
Augmented reality‐guided minimally invasive spine surgery and hepatectomy. A) Integration of 3D reconstruction of the spine with axial and sagittal CT images into the surgeon's field of view, resulting in a “see‐through” effect during spine surgery. B) Utilization of 3D reconstruction and navigation of crucial anatomical structures such as the hepatic artery (Red), portal vein (Green), and hepatic vein (Blue) during hepatectomy. Reproduced with permission. Copyright 2023, Elsevier and 2022, American College of Surgeons.^[^ ^89^, ^90^ ^]^

VR is a virtual environment that uses computers to conduct 3D simulations of real scenes, providing users with visual, auditory, tactile, and other sensory simulations so that they feel as if they are in the scene.^[^ ^83^ ^]^ Augmented reality (AR), developed from VR technology, is a hybrid technology that combines image recognition, virtual‐real integration, and human‐computer interaction to realize the interaction and reflection of the natural world by superimposing virtual scenes onto real scenes to realize the enhancement of reality.^[^ ^84^ ^]^ With the gradual advancements in VR/AR technologies, these techniques have been increasingly widely applied in the medical field, especially in clinical surgery for applications such as virtual surgery training and preoperative surgical path planning, and have attracted significant attention in orthopedics, liver surgery, neurosurgery, and other fields since the early 21st century (**Figure** **4**).^[^ ^85^, ^86^, ^87^, ^88^ ^]^

In contrast to the transparent virtual 3D models of organs, surgeons normally cannot observe the actual proximity to vital organs and vessels in the surgical field during a real operation, which increases the risk of accidental bleeding and organ injury. The development of VR/AR technology can provide surgeons with a pair of “perspective eyes” that can penetrate organs.^[^ ^85^ ^]^ Recent studies have indicated that AR technology significantly reduces surgical time and intraoperative blood loss, allowing advancements in precise liver surgery.^[^ ^90^, ^91^ ^]^ Accurate placement of pedicle screws in spinal surgery is challenging and often requires calibration during intraoperative fluoroscopy. AR can help surgeons perform highly accurate orthopedic procedures while minimizing radiation exposure to surgical personnel.^[^ ^92^, ^93^ ^]^ AR technology also plays an important role in neurosurgery. AR technology can facilitate maximal and accurate resection when determining the boundaries of intracranial tumors intraoperatively and allows preservation of adjacent neurovascular structures.^[^ ^94^, ^95^ ^]^ VR/AR techniques are currently a mature technology for hard tissues such as bones. However, the use of these techniques in procedures involving soft tissues such as the liver and gastrointestinal tract presents significant challenges, since 3D construction is mainly based on the preoperative CT scan examination, and large‐scale deformation of soft tissues during surgery leads to inaccurate positioning of organ structures.

### 3.4 Radionuclide‐Guided Surgery

Radionuclide imaging is a common technique in clinical nuclear medicine based on tracer technology.^[^ ^96^ ^]^ By using radionuclides (^99m^Tc, ^18^F, ^125^I, etc.) or their labeled chemical molecules as tracers, radiographic detection is performed to monitor the whereabouts of these tracers to reveal lesions. With recent advances in detector technology, real‐time radionuclide scintillation imaging can be achieved during surgery using mini‐gamma cameras, thereby enabling intraoperative navigation.^[^ ^97^, ^98^ ^]^

Radionuclide‐guided surgery (RGS) has been implemented in various surgical scenarios, mainly for SLN mapping and detection of tumors such as parathyroid tumors and gastrointestinal neuroendocrine tumors.^[^ ^99^, ^100^ ^]^ In SLN mapping, endoscopic injection of ^99m^Tc‐labeled tin colloid and intraoperative lymph scintillation imaging with a gamma camera have been performed to locate SLNs of esophageal cancer with an accuracy of 94%.^[^ ^101^ ^]^ This approach has also been widely used for the detection of SLNs in prostate cancer, melanoma, breast cancer, and stomach cancer.^[^ ^102^, ^103^, ^104^, ^105^ ^]^ Nevertheless, the limitations of RGS, such as additional radiation exposure for both patients and surgeons, the need for multiple scans, and complex scheduling issues related to the timing of radiopharmacodosing, need to be further addressed and refined.

### 3.5 Fluorescence‐Guided Surgery

Fluorescence‐guided surgery (FGS) uses exogenous fluorescent molecular probes to label tumors and important anatomical structures and employs fluorescence imaging systems to accurately position intraoperative lesions and guide surgeons in real time. FGS has recently emerged as a key technology in precision surgery, enabling accurate tumor imaging, SLN localization, visualization of important structures, and vascular perfusion imaging.^[^ ^106^ ^]^ To date, a series of near‐infrared (NIR) fluorescent imaging agents have been clinically approved, including fluorescein, MB, 5‐aminolevulinic acid (5‐ALA), and indocyanine green (ICG).^[^ ^107^ ^]^ The use of these fluorescent agents in surgery has increased significantly over the past few decades. For instance, 5‐ALA induces the accumulation of fluorescent porphyrin in glioblastomas, which has been used to guide complete tumor resection and improve progression‐free survival.^[^ ^108^ ^]^ The combination of folic acid and fluorescein isothiocyanate (folate‐FITC) has been used to target folic acid receptor α (FR‐α), which is highly expressed in ovarian cancer cells, for real‐time surgical visualization with tumor‐specific fluorescence imaging during ovarian cancer surgery.^[^ ^109^ ^]^

#### 3.5.1 Near‐Infrared‐I Fluorescence‐Guided Surgery

**Figure 5:**
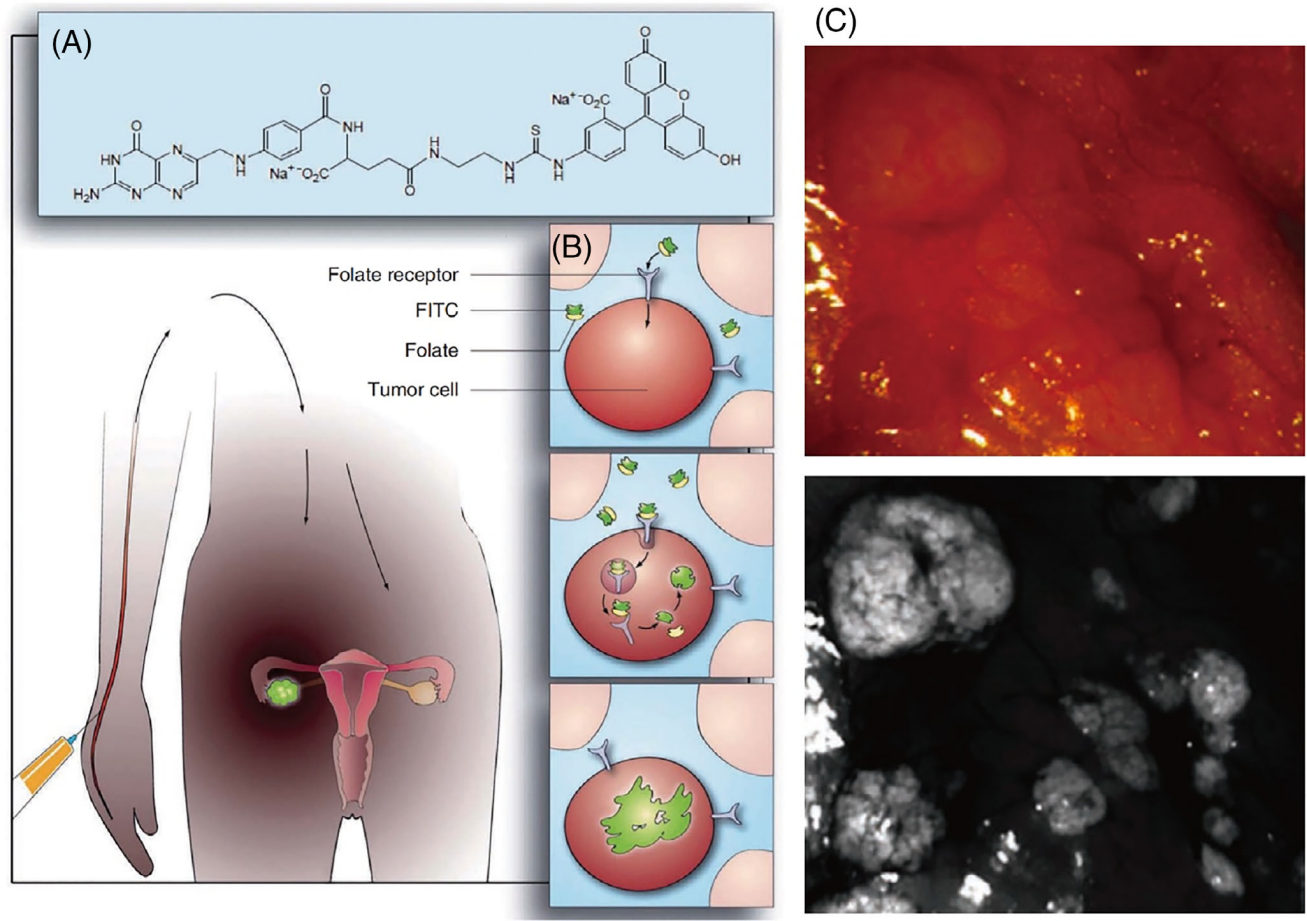
First‐in‐human intraoperative imaging in ovarian cancer by tumor‐specific folate receptor‐α targeting. A) Conjugation of folate with fluorescein isothiocyanate (FITC) via an ethylenediamine spacer, yielding folate‐FITC. B) Schematic representation illustrating the targeting of ovarian cancer by folate‐FITC. C) Color images accompanied by the corresponding tumor‐specific fluorescence image depicting representative lesions within the abdominal cavity. Reproduced with permission. Copyright 2011, Springer Nature.^[^ ^109^ ^]^

NIR (700–900 nm) fluorescence imaging has garnered considerable attention because of its excellent tissue penetration, high spatiotemporal resolution, and superior signal‐to‐background ratio.^[^ ^110^ ^]^ Thus, NIR fluorescent devices have been widely employed in endoscopy, laparoscopy, robotics, and open surgery.^[^ ^111^ ^]^ For example, Dr. Floris achieved unprecedented intraoperative ureteral NIR fluorescence imaging by injecting low doses of MB in patients to avoid iatrogenic ureteral injury.^[^ ^112^ ^]^ ICG‐based NIR fluorescence imaging is the most successful technique in liver surgery and can identify hepatocellular carcinoma with poorly demarcated margins in real time and with high sensitivity, and achieve intraoperative fluorescence cholangiography and vascular perfusion imaging to improve the accuracy of liver resection and surgical staging.^[^ ^113^, ^114^ ^]^ However, the current limitations of NIR fluorescence imaging in clinical applications, such as limited penetration depth, strong background signals, insufficient tumor specificity, and false positives, restrict its further promotion and application in clinical practice (**Figure** **5**).

#### 3.5.2 NIR‐II Fluorescence‐Guided Surgery

In the previous section, we discussed the wide range of clinical applications of NIR‐I (700^–^900 nm) FGS and described several approved NIR‐I fluorescence surgical‐navigation systems, including portable handheld, open surgery, and laparoscopic systems. More recently, a second form of NIR fluorescence (NIR‐II, 900–1880 nm) has been identified with advancements in optical technology.^[^ ^115^ ^]^ In comparison with NIR‐I fluorescence, NIR‐II fluorescence has a longer wavelength, resulting in increased absorption and reduced scattering of light by biological tissue, overcoming the interference from autofluorescence. Therefore, NIR‐II fluorescence imaging offers deeper tissue penetration, a lower background signal, and higher resolution.^[^ ^116^ ^]^ On the basis of these advantages, NIR‐II FGS has gradually become a popular topic in surgical‐navigation research since the concept was first proposed in 2009.^[^ ^117^ ^]^

In the ten years since 2009, many preclinical studies have significantly promoted the development of this field, including NIR‐II fluorescent probes, NIR‐II imaging systems, and different applications. In 2020, Tian et al. first applied NIR‐II fluorescence imaging techniques to liver resection using the FDA‐approved fluorescent dye ICG. They reported that intraoperative NIR‐II imaging‐based navigation improved the tumor‐detection rate and sensitivity.^[^ ^118^ ^]^ Furthermore, some preclinical studies have focused on novel navigation patterns of NIR‐II fluorescence imaging, providing new directions for future clinical applications. For example, Lin et al. performed lymph node dissection guided by multi‐channel NIR‐II imaging. By capitalizing on the higher resolution and penetration depth of NIR‐II, they were able to trace the removed lymph nodes with greater precision, while clearly displaying the surrounding important anatomical structures, such as blood vessels and ureters. This approach ensured that the adjacent vital tissues and organs were not damaged during complete and thorough lymph node dissection.^[^ ^119^ ^]^

**Figure 6:**
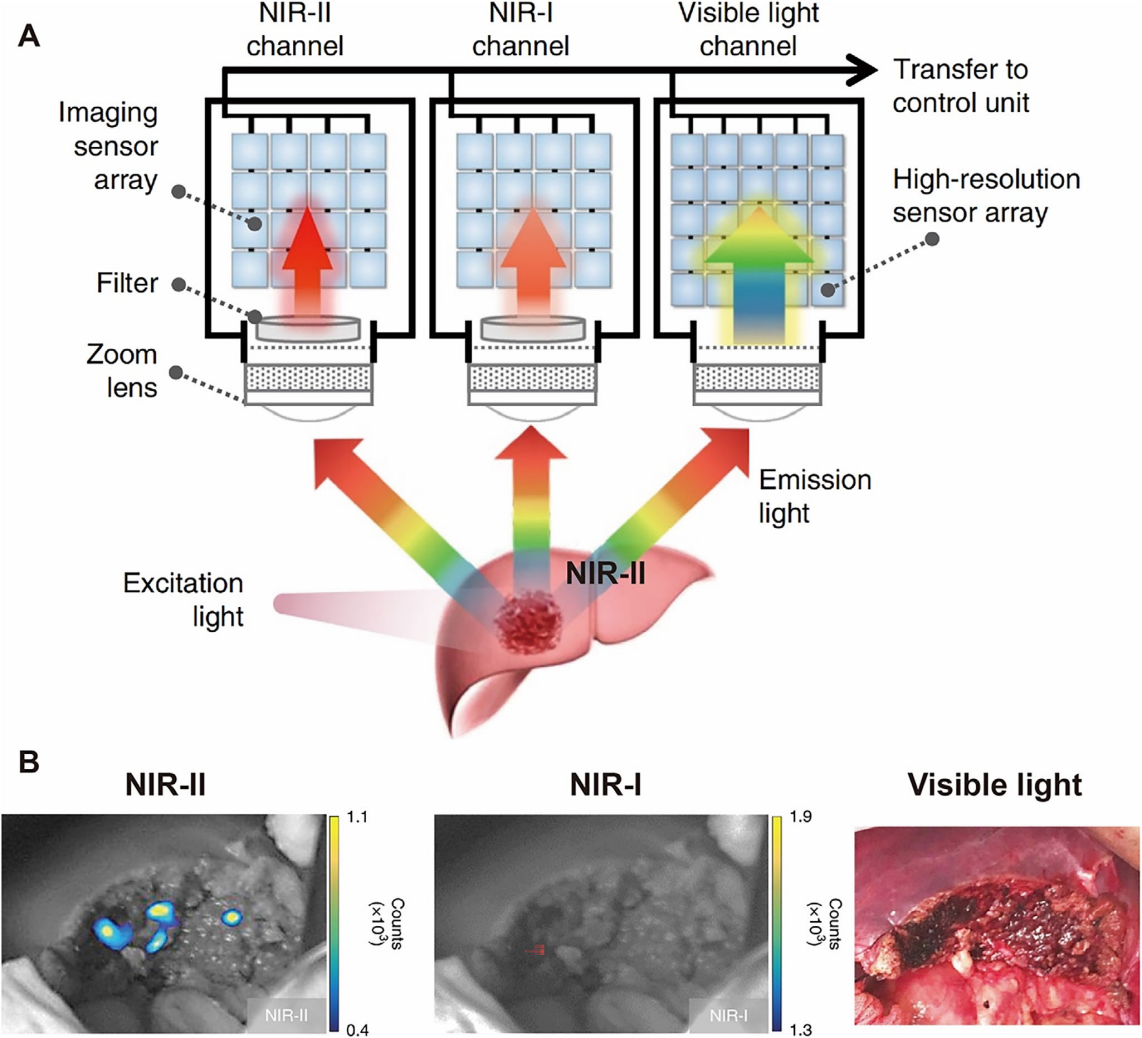
The visible and NIR‐Ι/II multispectral imaging instrument and its application in intraoperative image‐guided tumor resection. A) Schematic of the integrated visible and NIR‐I/II multispectral imaging instrument. B) NIR‐II imaging could detect fluorescence signals in the remaining tissue sections, while both the NIR‐I and visible light images did not reveal any signals or remaining tumor tissue. Reproduced with permission. Copyright 2020, Springer Nature.^[^ ^118^ ^]^

With the ongoing advancements in NIR‐II fluorescence imaging technology, numerous studies have demonstrated its superiority over NIR‐I and other traditional fluorescence imaging methods for in vivo imaging and surgical navigation.^[^ ^120^ ^]^ In recent studies, novel multifunctional NIR‐II fluorescent probes, including rare‐earth‐doped nanoparticles (RENPs), supramolecular polymer nanoparticles (SPNPs), and aggregation‐induced emission luminogens (AIEgens), have been successfully applied for in vivo imaging, offering new tools for accurate diagnosis and treatment of clinical diseases.^[^ ^121^, ^122^ ^]^ However, the field remains in its early stages of development, and many NIR‐II fluorescence materials have issues that cannot be overlooked. Long‐term research and preclinical assessments are required to develop a multifunctional, biocompatible, and optically superior probe and verify its potential for clinical transformation.^[^ ^123^ ^]^ Moreover, multimode optical instruments have to be developed for NIR‐II fluorescence imaging. The development of an NIR‐II fluorescent laparoscopic system is of great importance for future navigation in minimally invasive surgery (**Figure** **6**).

### 3.6 Photoacoustic Imaging

Photoacoustic imaging (PAI) has recently gained attention as a medical imaging tool because of its excellent optical molecular specificity and deep tissue imaging capabilities. PAI is based on the photoacoustic effect, which involves the conversion of absorbed light energy into sound energy. When the pulsed laser illuminates the target tissue, the chromophore in the tissue absorbs the pulsed light energy, undergoes a non‐radiative transition, and generates thermoelastic expansion, which then propagates outward in the form of an ultrasonic wave. The spatial distribution and functional characteristics of a target tissue can be further deduced on the basis of the distribution of acoustic signals in vivo.^[^ ^124^ ^]^ This approach combines the sharp contrast in optical imaging and the deep penetration in ultrasonic imaging with non‐ionizing excitation to achieve non‐damaging imaging. More importantly, various endogenous contrast agents in organisms, such as hemoglobin, melanin, lipids, and collagen, can be used to realize multifunctional imaging.^[^ ^125^ ^]^

**Figure 7:**
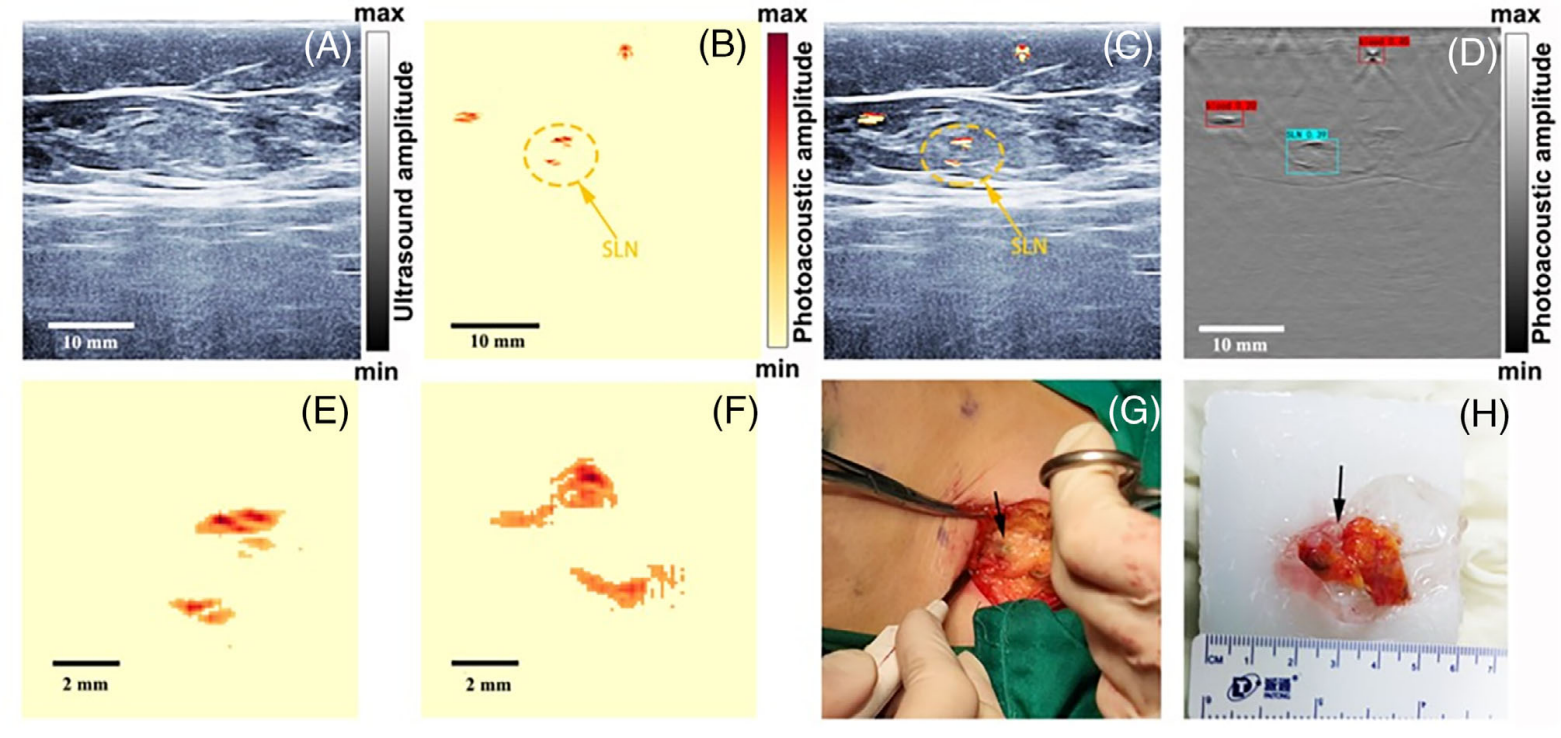
Sentinel lymph node mapping using a photoacoustic/ultrasound dual‐modality imaging system in patients with breast cancer. A–D) Representative images of in vivo PA/US imaging of SLNs for a patient with breast cancer. A) US image. B) PA image (SLN circled). C) Co‐registered PA/US image. D) The SLNs are automatically detected by the CNN network in the PA image. E‐H) Validation of the detected SLNs by removing the SLNs and ex vivo PA imaging. E) In vivo PA image of the SLNs. F) Ex vivo PA image of the resected SLNs in an agarose phantom. G) A photo of the stained lymph node (arrow) during surgery, which was guided by the photoacoustic (PA)/US imaging system. H) Photo of the ex vivo specimen (arrow points to the black‐stained lymph node). Reproduced with permission. Copyright 2023, The Optical Society.^[^ ^127^ ^]^

Since the 1990s, with advancements in lasers, ultrasonic detection technology, data‐acquisition systems, and computer technology, PAI has been applied in the biomedical field.^[^ ^124^ ^]^ For example, PAI has been shown to be particularly suitable for improving the imaging diagnosis of breast diseases owing to the lower ultrasonic scattering of breast tissue. The PAI device, which has been developed and tested on human volunteers, has been shown to be capable of visualizing the vascular system of the entire breast and detecting tiny breast cancer lesions owing to the strong inherent photoacoustic contrast between the aberrant vascular system and abnormally elevated hemoglobin levels at the tumor site.^[^ ^126^ ^]^ Using carbon nanoparticles, surgeons can also use PAI techniques to locate SLNs (**Figure** **7**).^[^ ^127^ ^]^ In addition, PAI can also be used for qualitative diagnosis of skin lesions, skin cancer, and inflammatory dermatitis to partly replace invasive examination methods such as skin biopsy and histopathology.^[^ ^128^ ^]^ PAI has also been used for obtaining angiograms of limbs, cerebral structural functional imaging, and musculoskeletal tissue imaging, and other applications, demonstrating its broad prospects as a burgeoning imaging technology.

PAI's strengths lie in its reasonable penetration depth, excellent spatial resolution, and the potential for real‐time imaging.^[^ ^129^ ^]^ As a result, PAI allows intraoperative mapping of tumor boundaries and tumor invasion to determine the resection margin and to help assess the presence of residual tumors after resection.^[^ ^130^ ^]^ In addition, PAI can play an important role in reducing iatrogenic injuries intraoperatively by improving the visualization of nerves, ureters, and blood vessels.^[^ ^131^, ^132^ ^]^ Multiple preclinical studies have been conducted using PAI for intraoperative navigation, indicating its extraordinary ability to accurately guide surgery and biopsy, which have been demonstrated in animal models.^[^ ^133^ ^]^ Therefore, surgical navigation can be optimistically estimated to be most valuable application scenario of PAI technology in the future.

### 3.7 Spectral Imaging Navigation Technology

Although conventional medical imaging techniques excel at revealing anatomical structures, they often fail to provide real‐time, detailed insights into tissue biochemistry and functional state during critical procedures such as surgery. Spectral imaging technologies have emerged as powerful solutions for this challenge. These techniques go beyond the limitations of human vision and conventional imaging by capturing and analyzing the unique “spectral fingerprints” generated when light interacts with matter. This allows non‐invasive, real‐time characterization of the deep molecular composition, chemical constituents, and physiological changes within tissues. The intrinsic contrast mechanism based on spectral signatures offers unprecedented potential for precisely delineating the boundaries between diseased and healthy tissues and identifying specific biomarkers during surgical interventions. This capability forms the technological foundation for true‐precision surgical navigation. This section explores two advanced spectral imaging modalities that demonstrate substantial potential for intraoperative guidance: RS and hyperspectral imaging (HSI). We examine their fundamental principles, unique advantages, recent clinical advancements, and ongoing challenges associated with their implementation.

#### 3.7.1 Raman Spectroscopy

Since the Raman effect was discovered in 1928, RS has become an important tool for detecting and analyzing material structures.^[^ ^134^ ^]^ By detecting molecular vibrations to speculate on the underlying chemical structure of a substance, RS can identify different chemical structures in complex samples, providing unique chemical fingerprints of specific molecules and materials. RS has also become a multifunctional tool for biomedical analysis because of its noninvasiveness, real‐time nature, and high specificity. In biological assessments, RS usually shows thousands of Raman spectral bands that can provide rich molecular information regarding biomolecules such as nucleic acids, proteins, and lipids and reflect the genotype, phenotype, and physiological state of tissues.^[^ ^135^ ^]^ Clinical diseases are often accompanied by malignant transformation of cells and tissues and notable biochemical changes; therefore, RS technology can be used to detect and quantify changes in molecular characteristics and is regarded as an excellent tool for disease diagnosis.

**Figure 8:**
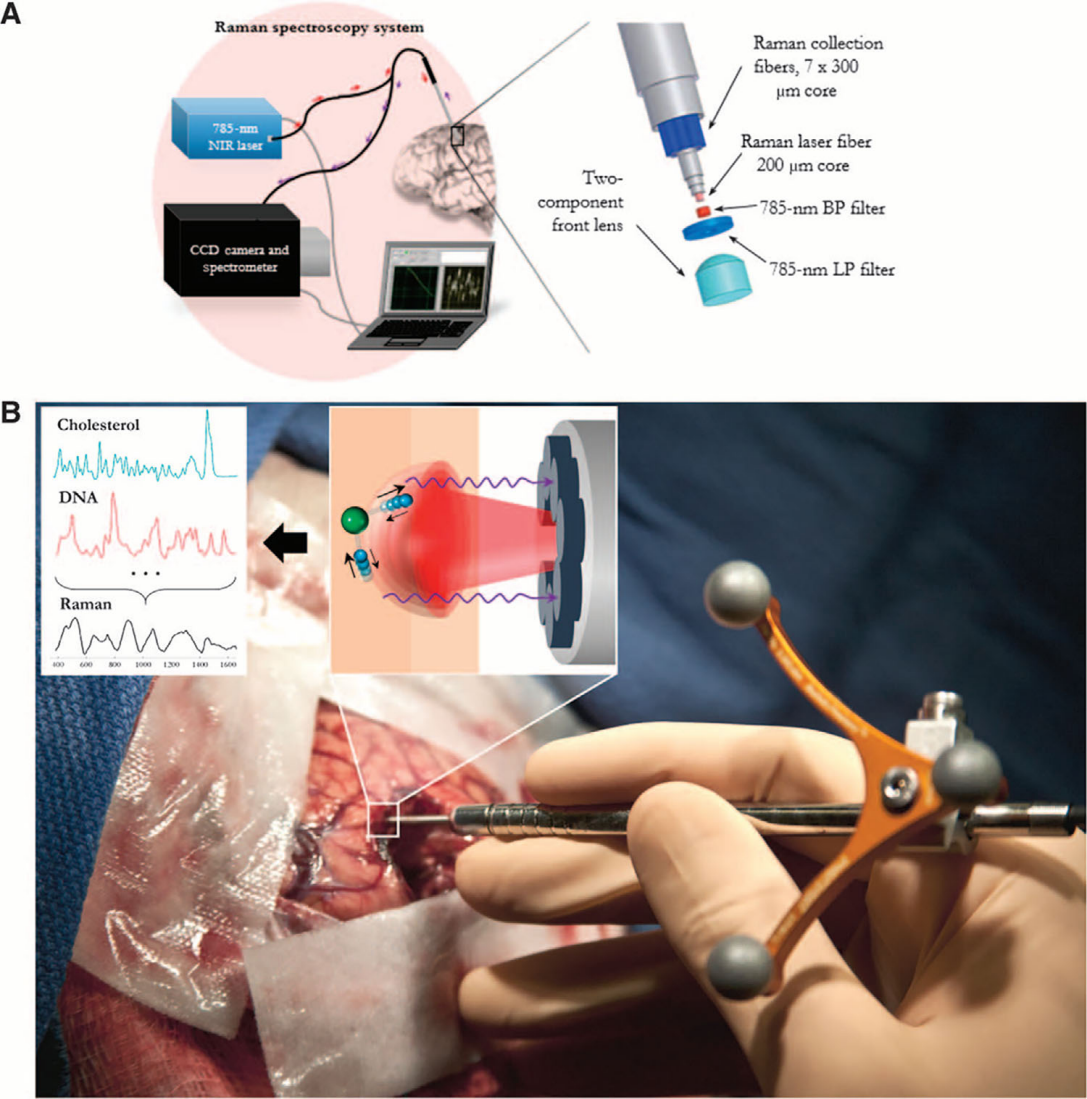
The handheld contact fiber optic probe for Raman spectroscopy in brain cancer detection. A) Experimental diagram with the 785‐nm NIR laser and the high‐resolution charge‐coupled device (CCD) spectroscopic detector used with the Raman fiber optic probe. B) The probe (Emvision, LLC) was used to detect brain tissue during the surgical procedure. The excitation of different molecular species, such as cholesterol and DNA, to produce the Raman spectra of cancer versus normal brain tissue. Reproduced with permission. Copyright 2015, American Association for the Advancement of Science (AAAS).^[^ ^140^ ^]^

RS has been used for intraoperative imaging to accurately distinguish the boundary between normal and diseased tissues and thereby guide precision surgery.^[^ ^136^ ^]^ Kast et al. demonstrated the ability of RS to distinguish white matter, gray matter, glioblastoma, and necrosis in frozen pathological sections, indicating its potential for enhancing pathological diagnosis during surgery.^[^ ^137^ ^]^ D'Acunto et al. developed an RS bioimaging technology to accurately distinguish the progression of chondromas from chondrosarcomas on the basis of the degree of collagen degradation, which has important clinical significance for intraoperative guidance.^[^ ^138^ ^]^ An intraoperative RS imaging trial was conducted in a patient with breast cancer who underwent partial mastectomy. To investigate the feasibility of intraoperative tumor margin evaluation, a clinical Raman system and fiber optic Raman probe were used to evaluate the surgical margin after tumor resection. RS showed an overall accuracy of 93% in comparison with the results of conventional histopathological assessments.^[^ ^139^ ^]^ For real‐time navigation, Jermyn et al. applied RS techniques to accurately distinguish normal brain tissue from dense cancer during brain surgery, and reported sensitivity and specificity values of 93% and 91%, respectively (**Figure** **8**).^[^ ^140^ ^]^

However, some limitations remain to be considered in the clinical application of RS.^[^ ^141^ ^]^ The technology needs to be improved, including improving the excitation light source, developing new RS probes to improve sensitivity, and shortening the time required for image acquisition.^[^ ^142^ ^]^ Moreover, in terms of application, other considerations include development of clinically feasible equipment, considering combinations of RS with other imaging methods to optimize imaging, establishing objective classification procedures for cell and tissue diagnosis based on Raman data, and further improving diagnostic accuracy by employing artificial intelligence.^[^ ^143^ ^]^ With further advancements in Raman technology, RS will undoubtedly be widely used in various fields of scientific research in the future.

#### 3.7.2 Hyperspectral Imaging

HSI is an advanced technology that simultaneously captures both spatial and spectral information of an image. With high spectral and relative spatial resolution, it provides 2D spatial data and one‐dimensional spectral data of the imaged object, revealing its chemical composition and physical morphology.^[^ ^144^ ^]^ Over the past few decades, HSI systems have been widely applied in various fields. Originally developed for mining and geological exploration, HSI systems are now extensively used in agriculture, mineralogy, astronomy, chemical imaging, and environmental studies.^[^ ^145^, ^146^, ^147^ ^]^ The rapid development of artificial intelligence and precision medicine has highlighted the potential of HSI in biomedicine.^[^ ^148^ ^]^ HSI can serve as a non‐invasive diagnostic tool and facilitate the evaluation of therapeutic approaches by measuring the reflection and absorption of light at different wavelengths. This technology provides information on various tissue components and their spatial distribution from the spectral features of each pixel in a hyperspectral image to identify diseased and healthy tissues.^[^ ^149^ ^]^

In 2013, Kiyotoki et al. reported the use of an HSI system to detect gastric cancer. In their study, 16 patients with gastroduodenal tumors were treated using endoscopic or surgical resection, and HSI within the visible light range (400–800 nm) was utilized in 14 of the 16 cases. The results demonstrated that the tumor samples were clearly distinguishable from the normal mucosa, with sensitivity and specificity of 78.8% and 92.5%, respectively.^[^ ^150^ ^]^ These findings indicate that HSI, as a surgical‐navigation technique, can play an important role in distinguishing healthy and diseased tissues, defining surgical boundaries, and enhancing the success rate of surgeries. In addition, HSI has been widely used to assess microcirculation changes in diabetic foot ulcers and predict clinical outcomes as well as in clinical fields such as retinal imaging, highlighting its substantial potential for biomedical applications.^[^ ^151^, ^152^ ^]^

However, as an emerging technology, HSI has certain limitations. The existing applications of HSI in the medical field have been largely limited to clinical trials, primarily because of the substantial amount of data in each medical hyperspectral image. Extracting useful information from these images requires extensive spectral dimension reduction and data calibration, correction, compression, and analysis.^[^ ^153^ ^]^ This process would require a considerable amount of time, posing a challenge in the biomedical field. Furthermore, developing methods to quickly acquire images of target objects in real time and effectively integrate spectral instruments and algorithms to rapidly deliver diagnostic results remains a challenge for engineers and scientists.^[^ ^154^ ^]^ We believe that continuous advancements and refinement of HSI will yield more extensive applications of this technology in the biomedical field, and that it will play an increasingly important role in clinical practice.

### 3.8 Cherenkov Luminescence Imaging

Cherenkov luminescence imaging (CLI) is an emerging optical imaging method that relies on Cherenkov radiation (CR). When high‐speed charged particles emitted from radionuclide decay move faster than light in a non‐vacuum medium, short‐wavelength electromagnetic radiation is emitted in the form of Cherenkov light (CL).^[^ ^155^ ^]^ CLI offers the advantages of high resolution, simplicity, and repeatability in optical imaging. Because of the abundance of available clinical probes, it possesses unique advantages for clinical translation.^[^ ^156^ ^]^ Therefore, the development and application of CLI have attracted increasing attention from researchers and surgeons.

**Figure 9:**
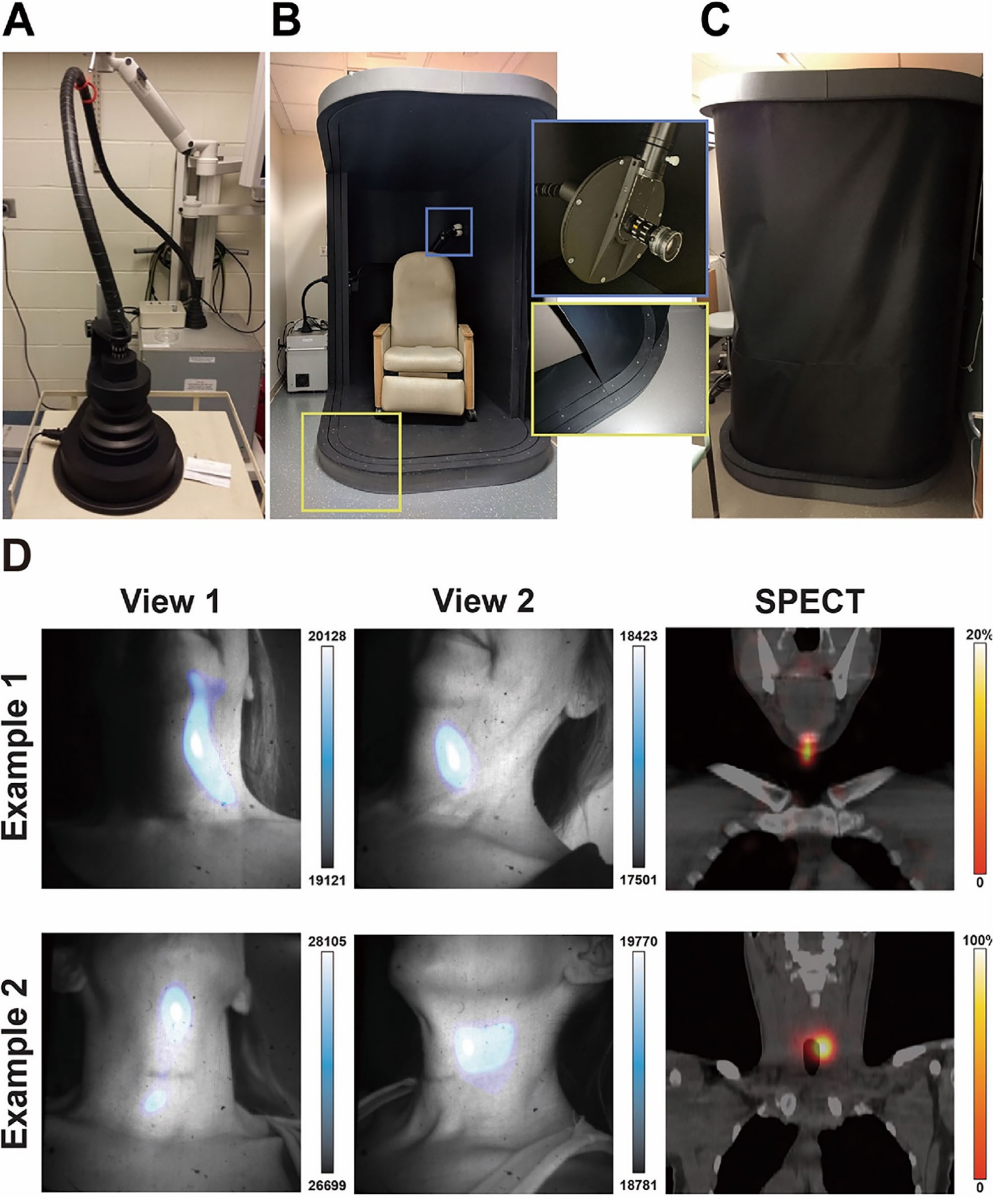
Clinical Cerenkov imaging system based on the Lightpoint fiberscope design and its application in the detection of ^131^I‐iodine in patients. A) The Cerenkov camera enclosed in a lead shielded box with a fiberscope connected to a preclinical enclosure. B) Clinical enclosure containing a reclining chair for patient imaging. The fiberscope is installed through the rear of the enclosure opposite the dual‐track shroud system. C) Both shrouds are deployed to prevent ambient light from reaching the patient during CLI. D) Example 1 and 2 display images from two angles depicting the CLI focal intensity, along with the corresponding single‐photon emission computed tomography (SPECT) images indicating uptake near the submental triangle. Reproduced with permission. Copyright 2022, Springer Nature.^[^ ^161^ ^]^

Many studies have described attempts to integrate the use of CLI in clinical practice. In 2009, Robertson et al. successfully used a highly sensitive CCD camera to realize CLI on tumor‐bearing nude mice by injecting the mice with ^18^F⁃ fluorodeoxyglucose (FDG), thus achieving the first dual‐mode PET/optical imaging of nuclide contrast media in vivo.^[^ ^157^ ^]^ Subsequently, Holland et al. performed CLI on mice with human epidermal growth factor receptor 2 (HER2)‐positive breast cancer using ^89^Zr‐desferrioxamine‐trastuzumab for preclinical surgical navigation.^[^ ^158^ ^]^ The entire process of tumor imaging and complete resection took less than 40 min, indicating that CLI can quickly and accurately locate and identify tumor boundaries and guide tumor resection. In addition, Thorek et al. used a subcutaneous injection of ^18^F‐FDG and found that CLI could visualize the lymph nodes even before the surgical field was exposed and could guide SLN resection.^[^ ^159^ ^]^ Furthermore, the development of Cherenkov's endoscopic imaging system (ECLI), which combined an optical fiber and CCD camera, could simulate laparoscopic guidance for tumor resection in tumor‐bearing nude mice, addressing the insufficient penetration depth of CLI and highlighting the potential for application of CLI in intraoperative navigation.^[^ ^160^ ^]^ In 2022, Pratt et al. conducted an epoch‐making clinical trial by establishing a clinical CLI fiberscope with a light‐proof enclosure to prove that CLI is a feasible and accurate method for cancer diagnosis (**Figure** **9**).^[^ ^161^ ^]^ We believe that CLI also shows great potential for clinical applications in surgical navigation. However, CLI has some limitations, such as weak optical signal intensity and susceptibility to interference. Several recent studies have proposed imaging strategies based on CLI, such as Cherenkov radiation energy transfer imaging (CRET) and secondary Cherenkov‐induced fluorescence imaging (SCIFI).^[^ ^162^, ^163^ ^]^ By using CL to excite specific fluorescent materials and generate detectable secondary optical signals, these strategies can effectively improve the fluorescence intensity and penetration depth, achieve a high signal‐to‐noise ratio, and show broad application prospects in the future.^[^ ^164^ ^]^ Undoubtedly, the use of surgical navigation through CLI will become increasingly common as relevant technologies continue to be updated.

### 3.9 Laser Speckle Contrast Imaging

When an optically rough surface is irradiated by a laser, the reflected or backscattered light interferes with itself owing to variations in the optical path to the imaging surface, producing a granular pattern consisting of dark and bright spots, which is known as a speckle.^[^ ^165^ ^]^ If moving scatterers, such as red blood cells, are present in the irradiated sample, the intensity of light at each pixel in the image changes over time, creating a dynamic speckle pattern. By analyzing the light signal after scattering and random interference, the velocity information of scattered particles, such as red blood cells, can be obtained, which is a fundamental principle of laser speckle contrast imaging (LSCI).^[^ ^166^ ^]^ LSCI offers several advantages such as high resolution, real‐time imaging, non‐contact operation, and simple equipment requirements. Owing to these advantages, it has attracted considerable attention from researchers and clinicians. By utilizing LSCI technology, the blood‐flow parameters of microcirculatory vessels can be obtained in real time, allowing for the evaluation of the structure of blood vessels, microcirculatory function, and metabolic activity. This information is of great importance for intraoperative guidance, scientific research, disease diagnosis, and analysis of therapeutic effects.^[^ ^165^ ^]^ LSCI has been widely used in basic research to achieve high‐resolution imaging of blood flow in various small animal organs, including the brain, skin, viscera, and retina. Several studies in mouse models have also demonstrated the ability of LSCI to detect microvascular remodeling and hemodynamic changes during wound‐healing angiogenesis.^[^ ^167^ ^]^

Clinicians have conducted preclinical and clinical trials to investigate the exceptional performance of LSCI in surgical guidance, precision diagnosis, and treatment, owing to its superior imaging capabilities. For example, Antonella et al. employed intraoperative LSCI for real‐time visualization of cerebral blood flow in cerebrovascular surgery, enabling intraoperative guidance and accurate operations.^[^ ^168^ ^]^ Emmanuel et al. used LSCI to detect intraoperative differences in the parathyroid vascular signals in patients undergoing thyroidectomy to reduce the incidence of postoperative hypocalcemia.^[^ ^169^ ^]^

**Figure 10:**
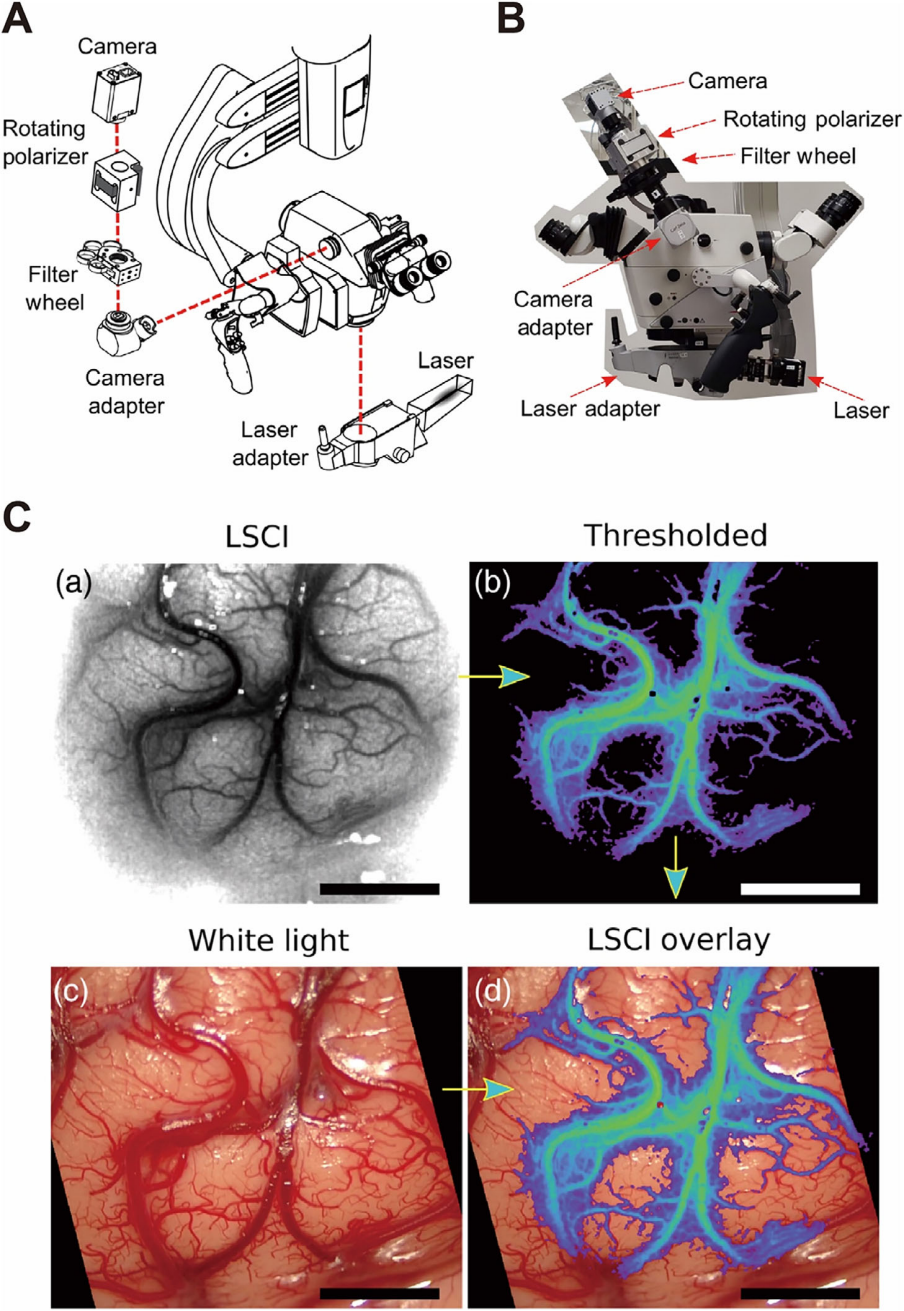
Continuous blood‐flow visualization with laser speckle contrast imaging during neurovascular surgery. A) Schematic of the Leica OH6 neurosurgical microscope outfitted with LSCI instrumentation. B) Photograph of the LSCI hardware in the operating room. C) Image‐processing steps to create the overlay of LSCI. (a) LSCI image with grayscale color map. (b) LSCI image with a threshold applied to display high‐flow blood vessels with median filter and pseudocolor applied. (c) White‐light image captured at the same time as LSCI image. (d) Thresholded pseudocolor LSCI image merged with the white‐light image to create an LSCI overlay image. Reproduced with permission. Copyright 2022, the International Society for Optics and Photonics.^[^ ^170^ ^]^

In comparison with other imaging techniques, LSCI can achieve millisecond temporal resolution and micron spatial resolution. Moreover, it is a contrast label‐free imaging technique that enables long‐term dynamic measurement of hemodynamics, making it a valuable tool for real‐time monitoring.^[^ ^170^ ^]^ With the ongoing research on improving key aspects of LSCI, including the signal‐to‐noise ratio, resolution, measurement accuracy, and imaging depth, as well as the continuous development of new LSCI device applications, including portable, endoscopic, and head‐mounted LSCI, the clinical application of LSCI can be extended from the body surface to procedures performed within the body, allowing for full coverage of surgical procedures (**Figure** **10**).^[^ ^171^, ^172^ ^]^

### 3.10 Confocal Laser Endomicroscopy

Confocal laser endomicroscopy (CLE) is an emerging microscopic imaging technology that provides high‐resolution imaging at the cellular level in vivo, making it a true “optical biopsy” technology.^[^ ^173^ ^]^ CLE is based on the imaging principle of laser scanning confocal microscopy (LSCM) and is often integrated into endoscopy, fluorescence microscopy, and computer image processing. Using ultraviolet or visible light to stimulate specific fluorescent probes such as fluorescein, CLE can be used to observe and detect the structure, molecules, and ions of living cells in real time and in situ. Owing to its high resolution, real‐time imaging, and non‐invasive nature, CLE has gradually become an important tool for real‐time diagnosis, guided biopsy, dynamic disease assessment, and follow‐up of cancer and other diseases.^[^ ^174^ ^]^

Fuchs et al. utilized acridine flavin‐assisted CLE for lung tissue imaging, which enabled detailed evaluation of cell structure and lung cancer with high accuracy (91%) and precise localization of tumors.^[^ ^175^ ^]^ Similarly, Julius et al. conducted a clinical study using CLE to perform intraoperative imaging in 12 cases of different brain tumors; the results showed that CLE is highly beneficial for providing high‐quality visualization of fine tissues and revealing hidden anatomical details.^[^ ^176^ ^]^ Yosuke et al. provided a comprehensive summary of the applications of CLE in abdominal surgery, particularly in gastrointestinal (GI) diseases, such as in situ detection of Barrett's esophagus, stomach tumors, inflammatory bowel disease, and colon polyps, as well as its usefulness in guiding uncertain biliary stenosis or pancreatic cyst puncture diagnosis.^[^ ^177^ ^]^ Further integration of CLE into the laparoscopic system has allowed surgeons to perform CLE examinations during laparoscopic surgery to identify the characteristics of microlesions at the cellular level, which can significantly enhances the visual perception of surgeons.^[^ ^178^ ^]^

**Figure 11:**
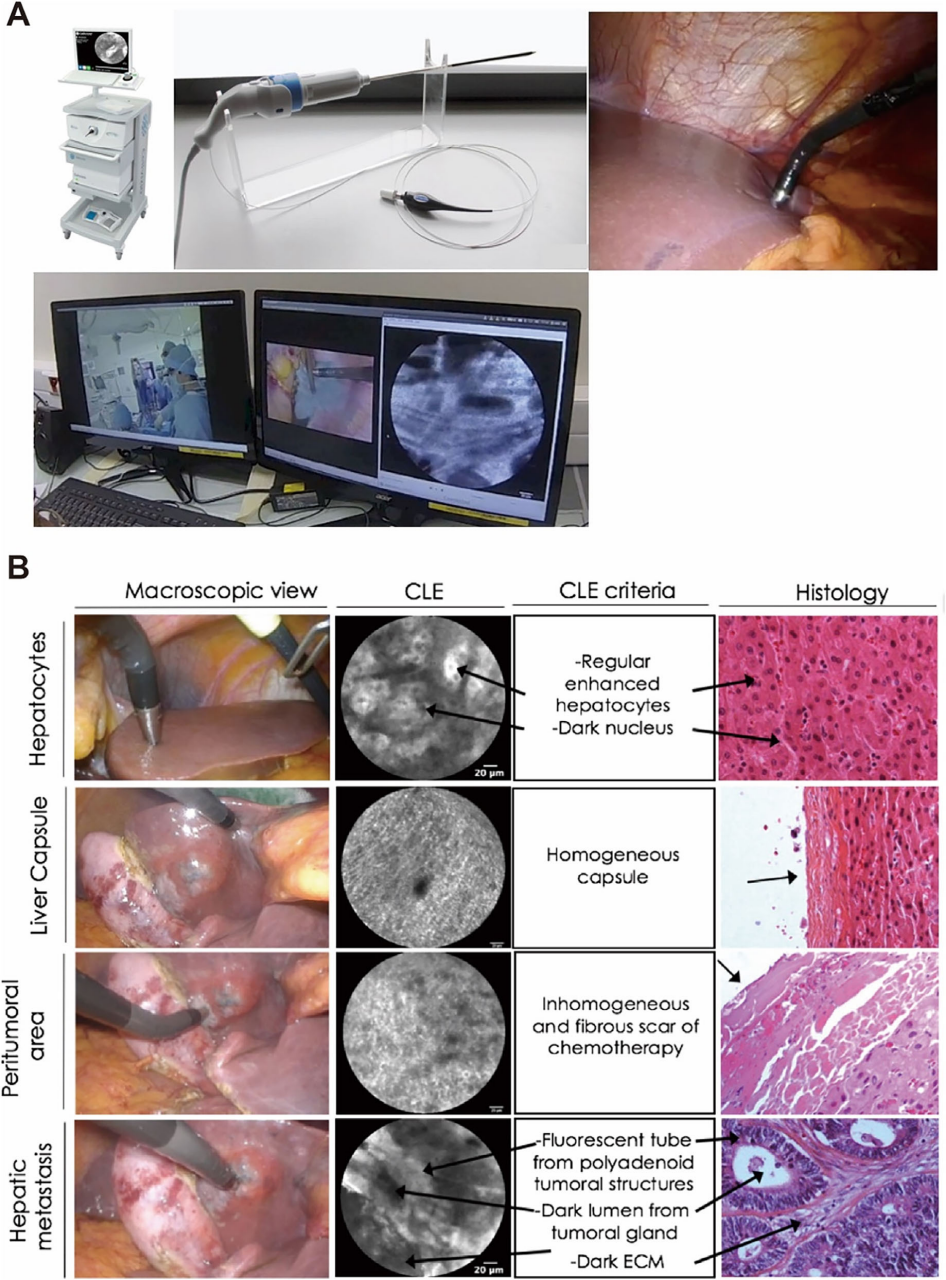
Intraoperative confocal laser endomicroscopy for real‐time tissue characterization during surgical procedures. A) Material A Cellvizio endomicroscopy systems and its specific practice in operation. Figures showing the operating room, laparoscopic view, and endomicroscopic view, from left to right. B) pCLE images from different liver tissues stained by topical application of ICG and correlation with corresponding histology. Reproduced with permission. Copyright 2019, Springer.^[^ ^178^ ^]^

However, CLE has several limitations that restrict its clinical application. For example, although CLE can allow 3D imaging, it may not display sufficient tissue depth or allow evaluation of the inherent and muscle layers. Additionally, factors such as respiratory motion and operational stability during the imaging process can cause the probe to lose stable contact with the lesion surface, which may limit the acquisition time of local images and affect the results (**Figure** **11**).^[^ ^179^ ^]^

## 4 Conclusions and Perspectives

Despite the advancements in surgical‐navigation technologies, several limitations of these technologies warrant attention. Acknowledging and address these limitations is crucial for continued improvement and widespread acceptance of these systems in the field of healthcare. One of the primary challenges faced by surgical‐navigation systems is achieving absolute accuracy, particularly in complex anatomical regions or dynamic surgical environments. Ensuring precision in such contexts is a persistent concern that requires continuous technological improvements. A potential method to solve this problem is to develop multi‐model detectors, which may significantly improve the accuracy of the navigation system. Second, implementation of surgical‐navigation technology has substantial economic implications. From equipment acquisition to maintenance and training expenses, the associated costs can be substantial, potentially acting as barriers to widespread adoption of these systems. Meanwhile, surgeons using surgical‐navigation systems may experience a learning curve. Extensive training is often required for effective application, and this learning process may affect the efficiency of the application of these systems in clinical practice. Streamlining training methods is crucial for reducing disruptions and enhancing proficiency. Increasing the acceptance of surgeons to different novel techniques is also a very important factor in promoting these techniques. In addition, the methods for nerve detection in clinical practice are limited. Therefore, a novel method is required in this field.

Furthermore, the dependence on technological infrastructure raises concerns about potential system failures, malfunctions, or interruptions during surgery. These issues are critical since they may compromise patient safety and affect surgical outcomes. Therefore, the development of robust fail‐safes and emergency plans is essential. Integrating navigation tools into the surgical workflow may cause interruptions and extend surgical times, potentially having a cascading effect on the overall efficiency and increasing the risk of complications. In this regard, maintaining a balance between the benefits of navigation and seamless workflow integration is crucial. In conclusion, while surgical navigation can undeniably enhance the precision and outcomes of various medical procedures, recognizing and actively addressing these challenges is necessary. By addressing accuracy limitations, managing costs, streamlining training, ensuring technological reliability, optimizing workflow integration, and adapting to specific patient nuances, this field can continue to evolve, providing enhanced navigational solutions for surgeons and improving patient care.

The clinical application of surgical‐navigation technologies also faces numerous challenges, including technology integration, API closure, and the use of real‐time image analysis software based on tools such as machine learning (ML) and artificial intelligence (AI). Integrating novel intraoperative navigation technologies with existing hospital information systems (HIS) presents significant challenges owing to the diverse and often outdated infrastructure in many hospitals.^[^ ^180^ ^]^ Closed APIs are a major obstacle to the adoption of intraoperative technologies because they restrict the interaction between new systems and existing tools. This limitation leads to fragmented workflows and reduces surgical efficiency. Promoting the use of open APIs is crucial for enhancing interoperability and fostering innovation in surgical‐navigation systems.^[^ ^181^ ^]^ Real‐time image analysis software leveraging ML and AI has become increasingly critical for surgical navigation. These technologies can be used to analyze images swiftly and accurately, and provide surgeons with crucial intraoperative insights. For example, ML algorithms can identify tumor margins with high precision, facilitating more accurate resection.^[^ ^182^ ^]^ However, challenges such as the need for large training datasets and ensuring algorithm reliability must be addressed for successful clinical integration.

The existing navigation technologies have both advantages and disadvantages. Future research in this field should focus on the advancement of more intelligent, minimally invasive, personalized, multimodal, and radiation‐free navigation techniques. This evolution will build on the foundation laid by classical optical tracking systems (e.g., infrared stereoscopic camera‐based navigation), which pioneered instrument localization and served as a precursor to modern real‐time optical navigation systems. This entails the utilization of intelligent control, automation, optical, electromagnetic, and ultrasonic technologies, and the integration of multiple positioning methods to achieve maximal accuracy in positioning information and enable real‐time intraoperative multi‐objective monitoring, thereby paving the way for novel directions in surgical navigation.

## Conflict of Interest

The authors declare no conflict of interest.

## Author Contributions

X.F. and X.L contributed equally to this work. H.L. and X.F. performed conceptualization; X.F., X.L., Q.X., G.C., J.C., and Z.S. performed visualization; H.L. and J.Q. performed supervision; X.F. and X.L. wrote original draft; X.F. and Y.F. wrote, reviewed, and edited the manuscript; P.A.K. performed language editing.
